# DFT Analysis of NO Adsorption on the Undoped and Ce-Doped LaCoO_3_ (011) Surface

**DOI:** 10.3390/ma12091379

**Published:** 2019-04-28

**Authors:** Xiaochen Li, Hongwei Gao

**Affiliations:** 1 Key Laboratory of Plant Resources and Chemistry in Arid Regions, Xinjiang Technical Institute of Physics and Chemistry, Chinese Academy of Sciences, Urumqi 830011, China; lixiaochen16@mails.ucas.ac.cn; 2 University of Chinese Academy of Sciences, Beijing 100049, China; 3 School of Life Science, Ludong University, Yantai 264025, China

**Keywords:** DFT calculations, LaCoO_3_ (011) surface, Ce doping, electron transfer, adsorption energy

## Abstract

Using the density functional theory (DFT) method, we investigated the adsorption of NO on the undoped and Ce-doped LaCoO_3_ (011) surface. According to our calculations, the best adsorption site is not changed after Ce doping. When the NO molecule is adsorbed on the perfect LaO-terminated LaCoO_3_ (011) surface, the most stable adsorption site is hollow-top, which corresponds to the hollow-NO configuration in our study. After the substitution of La with Ce, the adsorption energy of hollow-NO configuration is increased. For the perfect CoO_2_-terminated LaCoO_3_ (011) surface, it is found that Co-NO configuration is the preferential adsorption structure. Its adsorption energy can also be enhanced after Ce doping. When NO molecule is adsorbed on the undoped and Ce-doped LaO-terminated LaCoO_3_ (011) surface with hollow-NO configuration, it serves as the acceptor and electrons transfer from the surface to it in the adsorption process. On the contrary, for the Co-NO configuration of undoped and Ce-doped CoO_2_-terminated LaCoO_3_ (011) surface, NO molecule becomes the donor and loses electrons to the surface.

## 1. Introduction

Nitric oxide (NO), which is mainly produced from automotive vehicles and coal-fired power plants, has caused serious environmental pollution such as acid rain and photochemical smog. Therefore, it is highly urgent to investigate NO removal. To search for good catalytic materials for NO, a great deal of studies have been done [1,2,3,4,5]. In recent years, perovskites represented by ABO_3_ have been regarded as promising materials for NO removal owing to their high thermal stability, excellent oxidation activity, and low price [6,7,8,9]. Although there are a lot of NO removal methods, it is generally accepted that the oxidation of NO to NO_2_ is the key step [10]. According to the papers [11,12], lanthanum-based perovskite-type oxides (e.g., LaCoO_3_ and LaMnO_3_) exhibit excellent catalytic activity for NO oxidation at temperatures of 300–350 °C, especially LaCoO_3_. According to the research of Onrubia et al. [13], LaCoO_3_ achieves maximum NO conversion of 71% at 300 °C. Its catalytic activity can be enhanced substantially by modification with suitable transition metals and more and more researchers have an interest in the field [14,15,16]. Cerium (Ce), by general assent, is a good promoter in perovskite lattice due to its two different valence states—Ce^4+^ or Ce^3+^—high oxygen storage capability, and excellent redox properties [16,17,18,19]. For example, Wen et al. [14] reported that Ce-doped LaCoO_3_ samples exhibit good catalytic activity in the oxidation reaction of NO, with the highest conversion of 80% at about 300 °C. Studies have shown that after partial substitution with Ce, the change of oxidation state of Co ions and/or formation of vacancies contribute to the enhancement of catalytic activity [14,16,20].

In order to explore catalytic mechanism, some work has been done to investigate the reaction between adsorbed NO molecule and LaCoO_3_ surface by employing the density functional theory (DFT) method [12,21,22,23]. Liu et al. [21] found that the adsorption of NO on the LaCoO_3_ surface is accompanied by the formation of bonds such as Co-N, Co-O, and O-N. The surface reactivity of doped perovskites may have some differences from that of undoped perovskites. For example, adsorbed Bader charges of the Sr-doped LaCoO_3_ (100) surface are reduced by about 0.2 e when compared to the undoped cases [22]. Sun et al. [24] also reported that CO molecule is only weakly adsorbed on the pure LaFeO_3_ (010) surface compared to doped cases. Beyond that, oxygen vacancies can also affect the interaction between substrate and adsorbate, for instance, CO adsorbs more strongly on the oxygen defective CoO-terminated LaCoO_3_ (001) surface when compared to the perfect surface [25]. However, to the best of our knowledge, DFT calculations about the adsorption of NO molecule on the Ce-doped LaCoO_3_ surface have never been reported until now. Adsorption is often seen as the first step in the oxidation pathway of gases [26,27,28] and the most important step in studying the gas sensing properties of substrate [29,30,31]. Thus, studying adsorption behavior of NO is helpful not only to explore the reaction pathway of NO catalytic oxidation, but also to investigate the gas sensing mechanism of substrate. In this paper, the NO adsorption behavior on the undoped and Ce-doped LaCoO_3_ surface was studied systematically using the DFT method. What we mainly focused on was the influences of Ce substitution on adsorption properties (e.g., structures, energies, and electronic properties).

## 2. Computational Methods and Models

In our study, the adsorption properties of NO were investigated using the program DMol^3^ package of Materials Studio (MS, Accelrys, San Diego, CA, USA) 8.0 [32,33]. The exchange–correlation function was treated with the spin unrestricted generalized gradient approximation (GGA) with the Perdew–Burke–Ernzerhof (PBE) [34]. The DNP set, which stands for the double numerical plus polarization, was selected to describe the valence orbit of all the atoms. A hardness conserving semilocal pseudopotential, density functional semicore pseudopotential (DSSP) was applied. After the convergence test, a global orbital cutoff of 5.0 Å for atomic basis truncation and a k-point mesh of 3 × 3 × 1 for the Brillouin zone sampling were chosen and employed in all calculations. The results of the convergence test are presented in the Figures S1 and S2. In the geometry optimization progress, the convergence criteria with respect to the energy, force, and displacement were 1.0 × 10^−5^ Ha, 2.0 × 10^−3^ Ha/Å, and 5.0 × 10^−3^ Å, respectively. To gain electrons difference between adsorbed and isolated NO molecules, Mulliken Population Analysis (MPA) was paid extreme attention. After consulting literature regarding the NO adsorption on the surface of perovskite oxides [35,36], it was found that van der Waals (vdW) interaction function was not employed. Therefore, the vdW interactions were not considered in our research.

**Figure 1:**
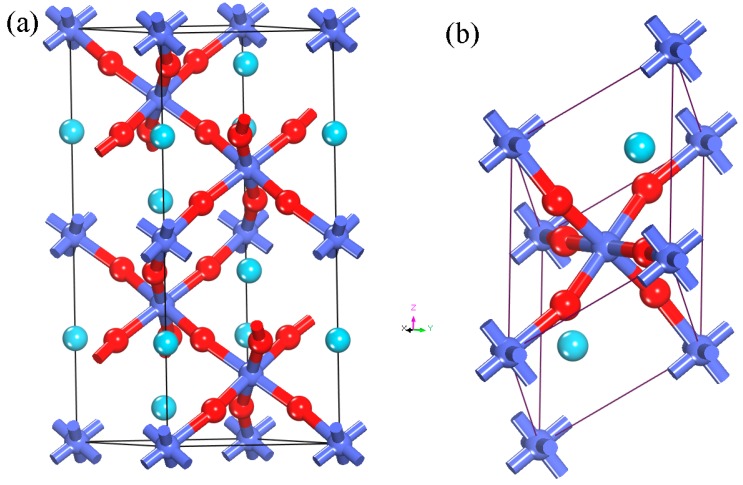
Schematic unit cell (**a**) and primitive cell (**b**) of LaCoO_3_. La atoms are shown in wathet blue, Co atoms in blue and O atoms in red. (For interpretation of the references to color in this figure caption, the reader is referred to the web version of this article).

The lattice parameters of rhombohedral LaCoO_3_ (see Figure 1a) are a = b = 5.4425 Å, c = 13.0929 Å, α = β = 90°, and γ = 120°, which are obtained from a prior experiment [37]. To simplify the calculations, we adopted the primitive cell of above-mentioned structure to conduct follow-up studies. Its lattice parameters are a = b = c = 5.3778 Å, α = β = γ = 60.8° (see Figure 1b). Before studying NO adsorption, we needed to determine relative stabilities of the (011), (111), and (010) surfaces, which are the frequently discussed surface slabs in perovskites such as LaFeO_3_, LaMnO_3_, and SrTiO_3_ [38,39,40,41,42]. The relative surface energy, E_surf_, can be determined using the following formula: E_surf_ = (E_slab_ − nE_bulk_)/2A. In this equation, E_slab_ and nE_bulk_ represent the energies of the relaxed LaCoO_3_ slab and an equal number (n) of bulk LaCoO_3_ atoms; A indicates the area of relaxed LaCoO_3_ slab; and the constant (2) reflects the fact that every slab has two surfaces [38,43]. By means of the formula, we obtained that surface energies of the (011), (111), and (010) surfaces are 1.334, 1.883, and 1.887 J·m^−2^, respectively. This is to say that the (011) is the most stable among the three surfaces. Therefore, the LaCoO_3_ (011) surface is employed in this study.

**Figure 2:**
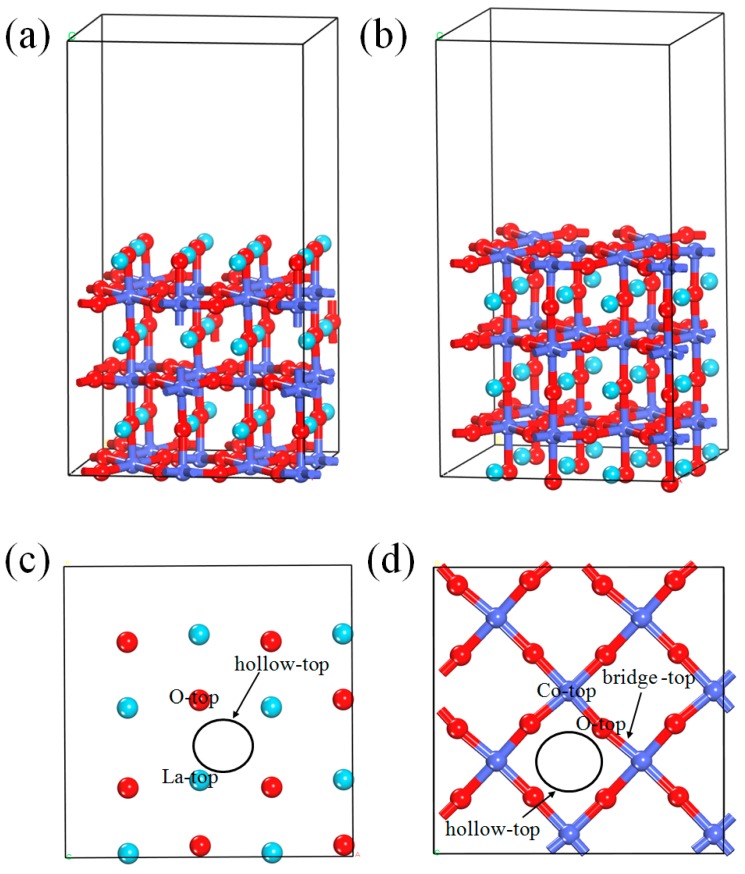
Side and top views of optimized LaCoO_3_ (011) surface: (**a**) the side view of LaO-terminated surface, (**b**) the side view of CoO_2_-terminated surface, (**c**) the top view of exposed (011) LaO termination, and (**d**) the top view of exposed (011) CoO_2_ termination.

The LaCoO_3_ (011) surface was modeled by cleaving the optimized primitive cell and adding a 15 Å vacuum layer to the unit cell. Then the 2 × 2 × 1 supercell with three LaO layers and three CoO_2_ layers was established and used to study the adsorption behavior of NO. There are LaO-terminated and CoO_2_-terminated surfaces along the (011) direction, whose side views are shown in Figure 2a,b, respectively. The top view of exposed (011) LaO termination and possible adsorption sites (La-top, O-top, and hollow-top) are displayed in Figure 2c,d, which similarly shows the top view of exposed (011) CoO_2_ termination and the corresponding Co-top, O-top, hollow-top, and bridge-top sites. Ce-doped species are modeled by substituting one La atom with one Ce atom. The calculated N-O bond length of free NO is 1.163 Å, which is slightly larger than the experimental value of 1.154 Å [44] and in excellent agreement with the previously calculated value (1.160 Å) [35,45]. In our study, the adsorption energy (E_ads_) is calculated by the following equation: E_ads_ = E_ad_ + E_sub_ − E_tot_, where E_ad_, E_sub_. and E_tot_ refer to the energies of the free NO molecule, the clean LaCoO_3_ (011) surface, and the LaCoO_3_ (011) surface with adsorbed NO molecule, respectively. By definition, a positive E_ads_ value indicates that the adsorbate–substrate system is stable, and the adsorption process is exothermic; a negative E_ads_ value indicates that the adsorbate–substrate system is unstable, and the adsorption process is endothermic. The bigger the value of E_ads_, the more stable the adsorption configuration.

## 3. Results and Discussion

### 3.1. NO Adsorption on the (011) Surface of Perfect LaCoO_3_

Both adsorption sites (La-top, Co-top, O-top, hollow-top, and bridge-top) and orientations of the NO molecule (N-down and O-down) may affect adsorption results. As previous DFT calculations have clarified that the N-down orientation is more benefit for NO adsorption [35,36,46], we focused our attention on N-down configurations. The article specifically discusses three initial adsorption configurations (La-NO, O-NO, and hollow-NO) for NO adsorption on the perfect LaO-terminated surface, and it also investigates four initial adsorption structures (Co-NO, O-NO, hollow-NO, and bridge-NO) for NO adsorption on the perfect CoO_2_-terminated surface.

#### 3.1.1. Calculation for the Perfect LaO-Terminated Surface

**Figure 3:**
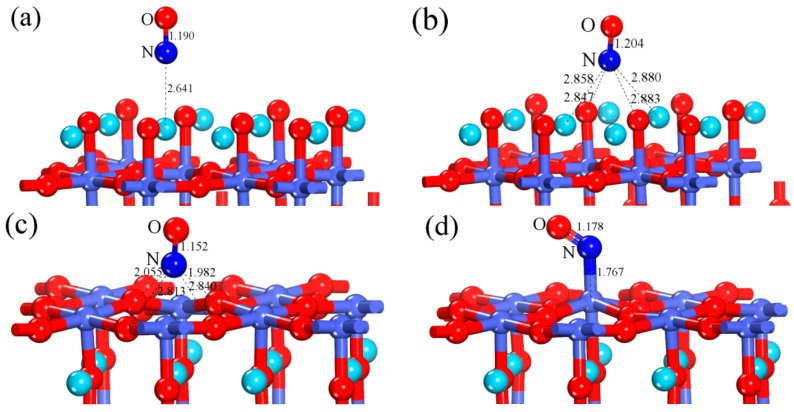
The optimized (**a**) La-NO and (**b**) hollow-NO configurations of the perfect LaO-terminated LaCoO_3_ (011) surface; the optimized (**c**) hollow-NO and (**d**) Co-NO configurations of the perfect CoO_2_-terminated LaCoO_3_ (011) surface.

After optimization of three adsorption structures mentioned above, we found an interesting phenomenon that although the adsorption model we built initially was the O-NO configuration, it became hollow-NO configuration. This means that the hollow-top site has stronger adsorption ability to NO molecule than O-top site. Therefore, only the hollow-NO and La-NO configurations, whose optimized structures are shown in Figure 3, are possibly stable structures. Next, we will focus on the two configurations and find out which structure is more suitable for NO adsorption. The E_ads_ is 0.471 eV for the La-NO and 0.593 eV for the hollow-NO, respectively. The values of E_ads_ are positive, indicating the two adsorption structures are stable and the adsorption processes are exothermic. Although both two adsorption structures may possibly occur, the NO molecule adsorbed preferentially on the surface with hollow-NO configuration. Its corresponding computation results, such as the optimized N-O bond length (d_N-O_), the N-O bond length difference between adsorbed and free NO molecules (△d_N-O_), the equilibrium distance (d) between the NO and the nearest substrate atom (La or O_surf_, the surface oxygen), the Mulliken charges (q_NO_), and the adsorption energy (E_ads_), are listed in Table 1.

**Figure 4:**
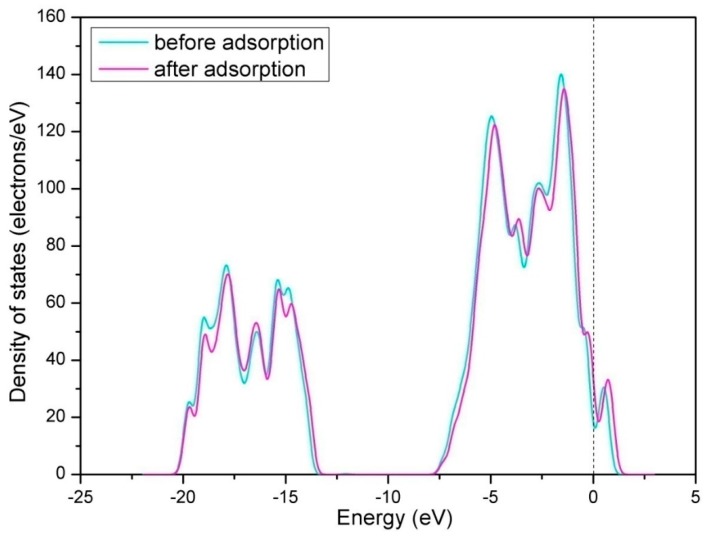
Total density of states of the perfect LaO-terminated LaCoO_3_ (011) surface before and after NO adsorption. Zero energy is the Fermi level, which is denoted as the vertical dotted line.

**Figure 5:**
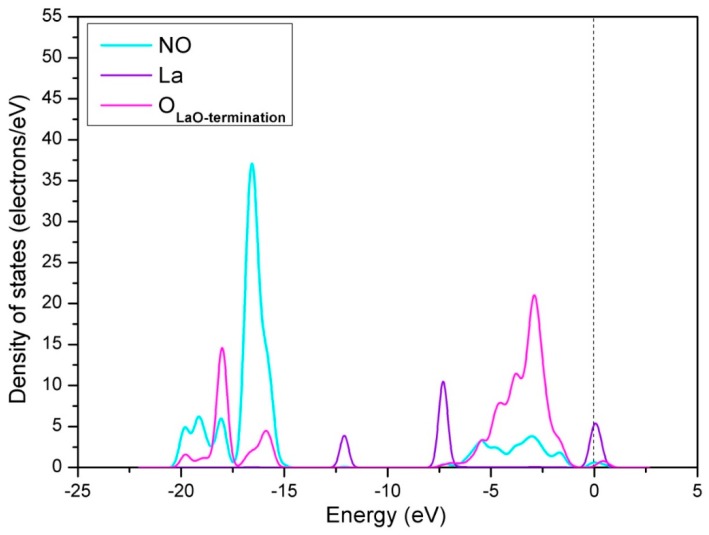
Density of states of adsorbed NO molecule, La and O atoms on the surface when NO is adsorbed on the perfect LaO-terminated LaCoO_3_ (011) surface with hollow-NO configuration.

For the hollow-NO configuration, the optimized N-O bond length (d_N-O_) is 1.204 Å, which is longer than the d_N-O_ of free NO (1.163 Å). The elongation of N-O bond has relation to the process of electron-transfer between adsorbed NO molecule and perfect LaO-terminated LaCoO_3_ (011) surface. To explain the electron transfer, the analysis of Mulliken charges for the hollow-NO configuration was employed (see Table 1). The calculated result indicates that the NO molecule serves as the acceptor and takes electrons from surface in the adsorption process, making it negatively charged with a net charge of 0.295e. Subsequently, the density of states (DOS) displayed in Figure 4 and Figure 5 were analyzed to clarify the interaction between adsorbed NO molecule and perfect LaO-terminated LaCoO_3_ (011) surface. Figure 4 shows the DOS of perfect LaO-terminated LaCoO_3_ (011) surface before and after NO adsorption. As can be seen from Figure 4, the peak shapes of DOS have a little change after adsorption, suggesting that the NO adsorption has only a little influence on the electronic structure of perfect LaO-terminated LaCoO_3_ (011) surface. In other words, there is a weak interaction between adsorbed NO and the surface when hollow-NO configuration occurs. The DOS of adsorbed NO molecule, La and O atoms on the surface are displayed in the Figure 5. From Figure 5, it can be seen that the orbits of NO molecule overlap with that of La and O atoms. More specifically, the NO hybridizes with both La and O atoms near −7.5 eV and the Fermi level. Thus, we can assert that the NO molecule can react with both La and O atoms on the surface when hollow-NO configuration occurs.

#### 3.1.2. Calculation for the Perfect CoO_2_-Terminated Surface

After optimization of the four initial adsorption structures mentioned above, it was found that the optimized O-NO and bridge-NO configurations were the same as the optimized Co-NO configuration, indicating that Co-NO configuration was more stable than O-NO and bridge-NO configurations. The optimized Co-NO and hollow-NO configurations are shown in the Figure 3. From Figure 3, we can see that for the hollow-NO configuration, the adsorbed NO molecule is detached from the surface, suggesting that the hollow-top is not the preferential adsorption site. Besides, the E_ads_ of hollow-NO configuration is 0.877 eV, which is lower than that of Co-NO configuration (1.302 eV). Based on the above analysis results, it is concluded that among the four adsorption structures we investigated, the Co-NO configuration is the most stable. The calculated result is consistent with the previous theoretical calculations that B-site metal has strong adsorption for some gas (e.g., CO and O_2_) [24,47,48]. Next, we will discuss the Co-NO configuration in detail. Its corresponding parameters including d_N-O_, △d_N-O_, d, q_NO_, and E_ads_ are listed in Table 1. It is learned from Table 1 that the optimized equilibrium distance (d) between the NO molecule and adsorbed Co site is 1.767 Å, which is short enough to form Co-N bond. The N-O bond length (d_N-O_) of adsorbed NO is slightly longer than that of isolated NO (1.178 Å against 1.163 Å). This indicates a weakening of the N-O bond when adsorption takes place [49]. As with Section 3.1.1, the electron transfer process resulted in the elongation of the N-O bond. According to Table 1, the NO molecule serves as the donor and loses 0.126e to surface in the adsorption process.

**Figure 6:**
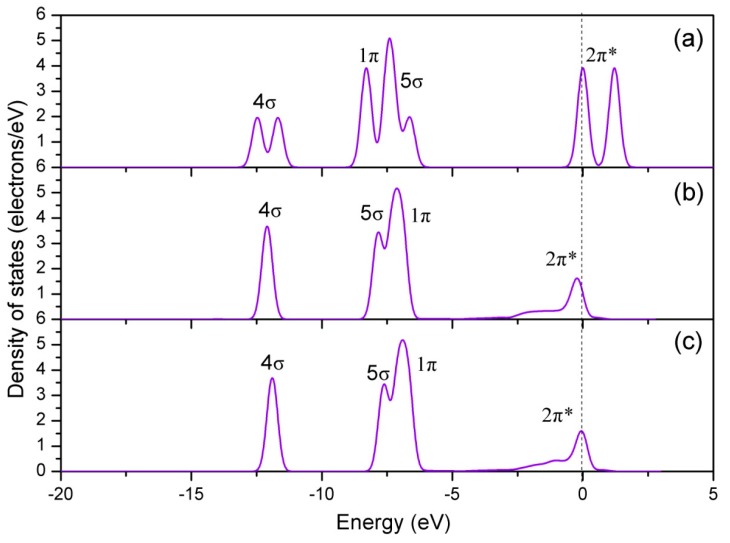
Density of states of the free and adsorbed NO: (**a**) Free, (**b**) perfect CoO_2_-terminated LaCoO_3_ (011) surface, and (**c**) Ce-doped CoO_2_-terminated LaCoO_3_ (011) surface.

**Figure 7:**
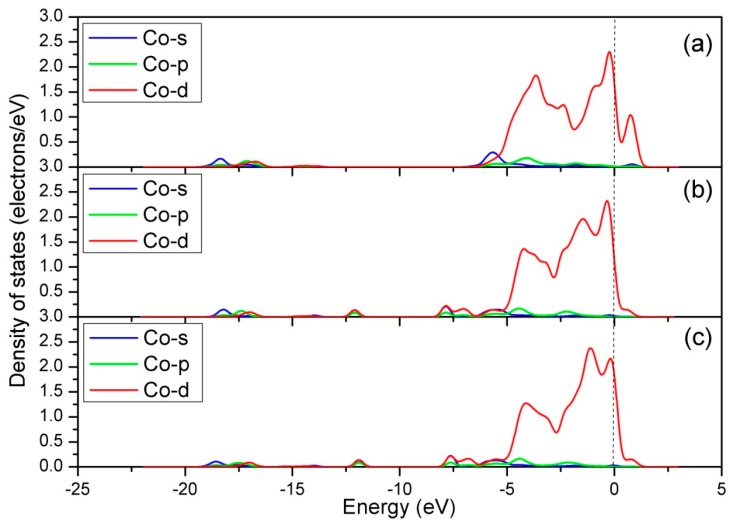
Density of states of the adsorbed Co site: (**a**) Before NO adsorption for the perfect CoO_2_-terminated LaCoO_3_ (011) surface, (**b**) after NO adsorption for the perfect CoO_2_-terminated LaCoO_3_ (011) surface, and (**c**) after NO adsorption for the Ce-doped CoO_2_-terminated LaCoO_3_ (011) surface.

**Figure 8:**
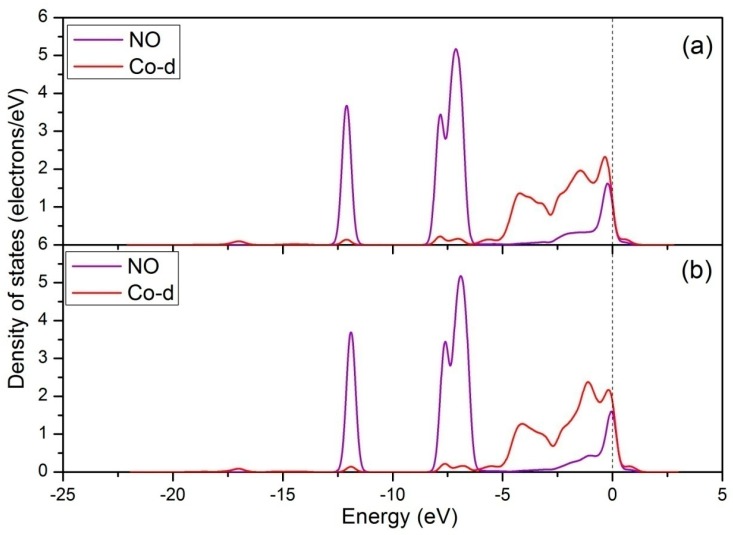
Density of states of the NO molecule and Co-d orbit: (**a**) For NO adsorption on the perfect CoO_2_-terminated LaCoO_3_ (011) surface, (**b**) for NO adsorption on the Ce-doped CoO_2_-terminated LaCoO_3_ (011) surface.

Next, the DOS was calculated to gain further insights into the bonding mechanism of Co-NO configuration. It is well known that the electron configuration of NO is K K($\sigma_{2S}$)^2^($\sigma_{2S}^{*}$)^2^(π_2px_)^2^(π_2py_)^2^(σ_2pz_)^2^($\pi_{2\mathrm{px}}^{*}$)^1^($\pi_{2\mathrm{py}}^{*}$)^0^ [50]. There are six electrons in the σ orbits, four electrons in the 1 π orbits, and one electron in the 2 π^*^ antibonding orbit. The DOS of free NO molecule is shown in Figure 6a, where each individual peak represents the interacting molecular orbits of NO. These orbits are termed as 4 σ, 1 π, 5 σ, and 2 π^*^, and appear at approximately −12.5, −8.5, −7.0, and 0 eV, respectively. Figure 6b shows the DOS of NO molecule adsorbed on the Co-top site of perfect CoO_2_-terminated LaCoO_3_ (011) surface. It is clear that the variation in shape and position of 5 σ and 2 π^*^ orbits is significant after adsorption, which mainly results from the 5 σ donation and 2 π^*^ back donation. The donation–back–donation process is that the NO 5 σ orbit donates electrons to the metal d-bands, while the metal d-bands donate them back to the NO 2 π^*^ orbit [51,52]. As a result, the 1 π orbit is slightly higher in energy than the 5 σ orbit. After this, we analyzed the DOS of Co atom before and after NO adsorption (Figure 7). It is clearly seen that the s and p orbits of Co atom show slight changes in the energy region of 5 σ, and the d orbit shows obvious changes in the energy region of 5 σ of adsorbed NO molecule and near the Fermi level. The above calculation results show that all orbits of Co atom participate in the Co-N bond formation. The intense reaction between adsorbed NO molecule and Co-d orbit is confirmed by Figure 8a, showing the DOS of interaction between them. From Figure 8a, it is seen that there is a significant hybridization between the NO molecule and the Co-d orbit, certifying that the Co-d orbit plays an important role in the bonding of Co-NO configuration.

### 3.2. NO Adsorption on the (011) Surface of Ce-Doped LaCoO_3_

Adsorption results of Ce-doped LaCoO_3_ (011) surface may be affected by doping sites. Therefore, we first tried to calculate the E_ads_ when Ce was doped into different layers. For the Ce-doped LaO-terminated surface, we took the hollow-NO configuration to calculate the E_ads_ when Ce was doped into in the first, third, and fifth layer of the perfect LaO-terminated surface. The calculated E_ads_ of the first, third, and fifth layer are 0.849 eV, 0.621 eV, and 0.609 eV, respectively—implying that when Ce is doped into the first layer, it has the best adsorption ability. Therefore, we gave attention to the simulation model that Ce was doped into the first layer. Similarly, for the Ce-doped CoO_2_-terminated surface, the Co-NO configuration was chosen to calculate the E_ads_ when Ce was doped into in the second, fourth, and sixth layer. The calculated E_ads_ are 1.380 eV, 1.252 eV, and 1.240 eV, respectively. Therefore, we will concentrate our efforts on the structure formed by doping Ce into the second layer.

#### 3.2.1. Calculation for the Ce-doped LaO-Terminated Surface

**Figure 9:**
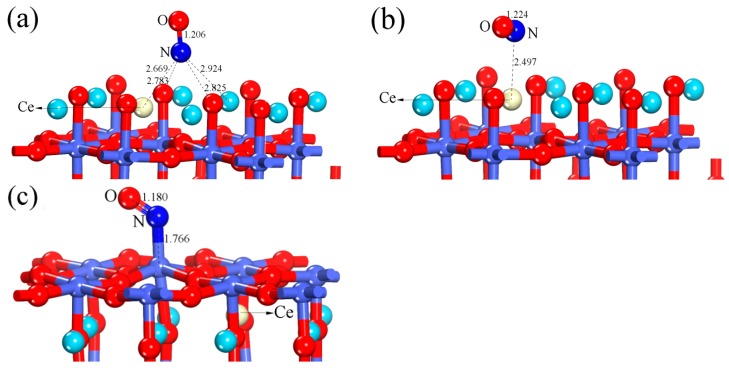
The optimized (**a**) hollow-NO and (**b**) Ce-NO configurations of the Ce-doped LaO-terminated LaCoO_3_ (011) surface; the optimized (**c**) Co-NO configuration of the Ce-doped CoO_2_-terminated LaCoO_3_ (011) surface.

According to the above discussion (Section 3.1), it has been known that the hollow-NO configuration is the most stable structure when NO is adsorbed on the perfect LaO-terminated LaCoO_3_ (011) surface. To explore the effects of Ce doping on adsorption properties, the hollow-NO configuration of Ce-doped LaO-terminated LaCoO_3_ (011) surface, whose optimized structure and corresponding parameters are displayed in Figure 9a and Table 1, respectively, was selected as a research object and discussed in detail. After Ce doping, the E_ads_ and q_NO_ for hollow-NO configuration increased by 0.256 eV and 0.022e, respectively. The N-O bond length of adsorbed NO molecule is 1.206 Å. This makes little difference in N-O bond length (0.002 Å) compared with the undoped case. When NO is adsorbed on the Ce-doped LaO-terminated LaCoO_3_ (011) surface, Ce-top is considered to be a possible adsorption site due to the fact that atoms tends to be attached to the defects [53]. The optimized Ce-NO configuration and corresponding parameters are displayed in Figure 9b and Table 1 respectively. From our calculations, elongation of N-O bond for the Ce-NO configuration is longer than that for the hollow-NO configuration of Ce-doped LaO-terminated LaCoO_3_ (011) surface. The number of electrons transferred from the surface to NO increased by 0.054e compared to that for the hollow-NO configuration of Ce-doped LaO-terminated LaCoO_3_ (011) surface. However, the E_ads_ of Ce-NO configuration (0.797 eV) is lower than that of hollow-NO configuration (0.849 eV). The above calculated results show that for the Ce-doped LaO-terminated LaCoO_3_ (011) surface, hollow-NO configuration is still the most favorable adsorption structure. In this structure, the introduction of Ce can cause the increase in adsorption energy and electron transfer numbers, making the Ce-doped surface have stronger adsorption ability to NO molecule.

**Figure 10:**
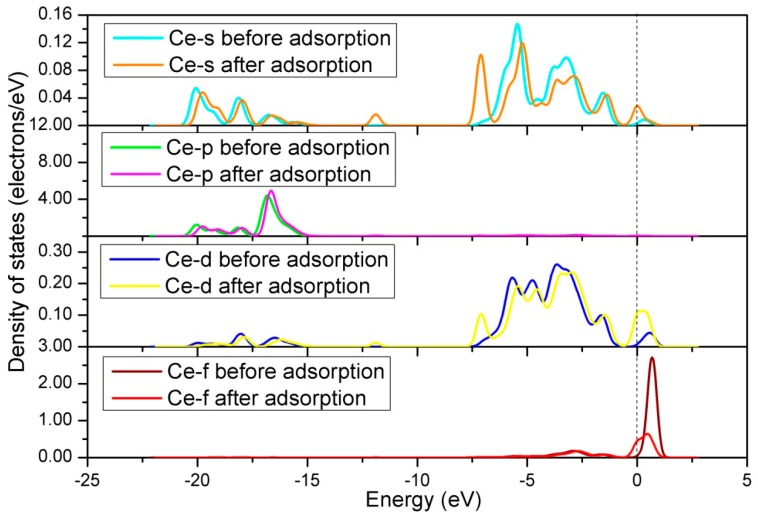
Density of states of Ce atom before and after NO adsorption for the Ce-doped LaO-terminated LaCoO_3_ (011) surface.

**Figure 11:**
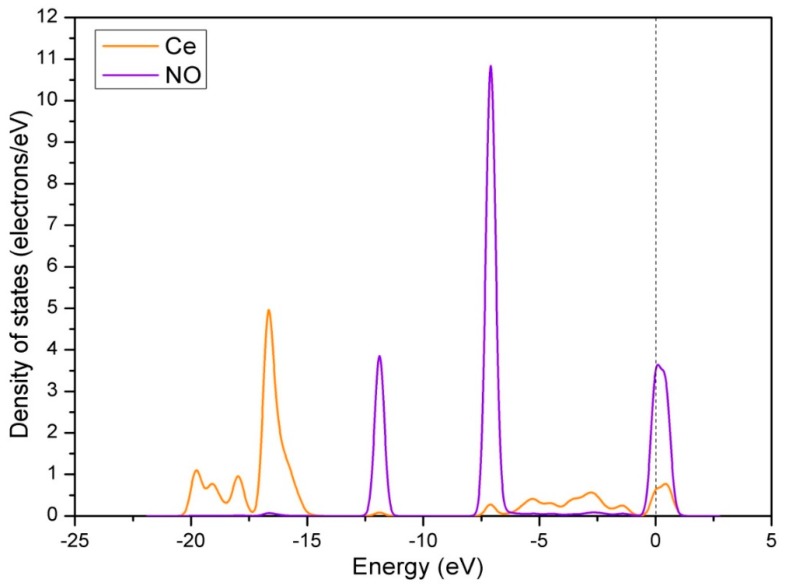
Density of states of adsorbed NO molecule and Ce atom when NO is adsorbed on the Ce-doped LaO-terminated LaCoO_3_ (011) surface with hollow-NO configuration.

The influences of Ce doping on the interaction between adsorbed NO and the adsorbate were obtained by the analysis of DOS. Firstly, we calculated the DOS of Ce atom on the surface before and after adsorption, as shown in Figure 10. From Figure 10, we can get main results as follows: (1) The s, d, and f orbits have a noticeable change near Fermi level; (2) the p orbit has a slight change near −17.0 eV. This means that all orbits of the Ce atom participate in the reaction between the NO and the surface. Figure 11 shows the DOS of adsorbed NO molecule and Ce atom. We can clearly see that there is a strong hybridization between the NO molecule and the Ce atom, certifying that the Ce doping has an active influence on the interaction between adsorbed NO and the surface.

#### 3.2.2. Calculation for the Ce-doped CoO_2_-Terminated Surface

The optimized Co-NO configuration that NO molecule is attached to the Co-top site of the Ce-doped CoO_2_-terminated LaCoO_3_ (011) surface is shown in Figure 9c, and the corresponding parameters are listed in Table 1. As seen from Table 1, the bond length of NO is elongated to 1.180 Å after adsorption on the Ce-doped CoO_2_-terminated LaCoO_3_ (011) surface, which is minimal change when compared to undoped case (1.178 Å). The E_ads_ of Co-NO configuration has been increased by 0.078 eV after the introduction of Ce. The number of electrons transferred from the NO molecule to the surface is 0.104e, which is a slight decline compared with the undoped case (0.126e).

For the Co-NO configuration of Ce-doped CoO_2_-terminated LaCoO_3_ (011) surface, the DOS of adsorbed NO and Co atom are displayed in Figure 6c and Figure 7c respectively. As shown in Figure 6c, the 5 σ and 2 π^*^ orbits of NO molecule show slight changes when compared to the case for NO molecule adsorbed on the perfect CoO_2_-terminated LaCoO_3_ (011) surface, which may be attributed to the difference in the number of electrons transferred. Comparing Figure 7b,c, it is found that the DOS of adsorbed Co site is almost unchanged before and after Ce doping. Furthermore, it is obvious from Figure 8 that the orbital hybridization between adsorbed NO molecule and Co-d orbit for NO adsorption on the Ce-doped CoO_2_-terminated surface is almost the same as that for NO adsorption on the perfect CoO_2_-terminated surface. All the calculated results indicate that the introduction of Ce into CoO_2_-terminated LaCoO_3_ (011) surface does not change the bonding mechanism of the Co-NO configuration.

## 4. Conclusions

In this work, using the DFT method, the adsorption reactions of NO on the undoped and Ce-doped LaCoO_3_ (011) surfaces were carried out and the effects of Ce doping on the adsorption reactions were analyzed. According to our calculations, when NO is adsorbed on the perfect LaO-terminated and CoO_2_-terminated LaCoO_3_ (011) surface, the most favorable adsorption structure is hollow-NO and Co-NO configuration, respectively. For the hollow-NO configuration of perfect LaO-terminated LaCoO_3_ (011) surface, the NO molecule serves as the acceptor, and about 0.295e electrons are transferred from the surface to it. The calculated DOS shows that adsorbed NO molecule can react with both La and O atoms on the surface. As for the Co-NO configuration of perfect CoO_2_-terminated LaCoO_3_ (011) surface, the NO molecule becomes the donor and loses 0.126e to the surface in the adsorption process. The formed Co-N bond is mainly dominated by the hybridization between adsorbed NO and Co-d orbit.

When NO molecule is adsorbed on the Ce-doped LaCoO_3_ (011) surface, the most suitable structure is hollow-NO configuration for the LaO-terminated surface and Co-NO configuration for the CoO_2_-terminated surface. Calculated results demonstrate that the best adsorption structure is not changed after Ce doping. For the hollow-NO configuration of Ce-doped LaO-terminated LaCoO_3_ (011) surface, the Ce doping can result in the increase of adsorption energy and the number of electrons transferred from the surface to the NO. It can also have an active influence on the interaction between adsorbed NO and the surface. As for the Co-NO configuration of Ce-doped CoO_2_-terminated LaCoO_3_ (011) surface, although the number of electrons transferred from the NO molecule to the surface slightly reduce when compared to the perfect CoO_2_-terminated LaCoO_3_ (011) surface, there is an increase in adsorption energy. In addition, the analysis of DOS has suggested that the bonding mechanism of Co-NO configuration is not affected by Ce doping. The interaction between adsorbed NO and Co-d orbit still plays a predominate role in the formation of Co-N bond.

## Figures and Tables

**Table 1:** Summary of the results of the NO molecule adsorbed on undoped and Ce-doped LaCoO_3_ (011) surface. The properties listed are as follows: The N-O bond length (d_N-O_), the N-O bond length difference between adsorbed and free NO molecules (△d_N-O_), the equilibrium distance (d), Mulliken charges (q_NO_), and the adsorption energy (E_ads_).

Configuration	d_N-O_	Δd_N-O_ (Å)	d (Å)	q_NO_ (e)	E_ads_ (eV)
Perfect LaO-terminated surface (hollow-NO)	1.204	0.041	2.847	−0.295	0.593
Perfect CoO_2_-terminated surface (Co-NO)	1.178	0.015	1.767	0.126	1.302
Ce-doped LaO-terminated surface (hollow-NO)	1.206	0.043	2.669	−0.317	0.849
Ce-doped LaO-terminated surface (Ce-NO)	1.224	0.061	2.497	−0.371	0.797
Ce-doped CoO_2_-terminated surface (Co-NO)	1.18	0.017	1.766	0.104	1.380

## Supplementary Materials

The following are available online at https://www.mdpi.com/1996-1944/12/9/1379/s1, Figure S1: Convergence test image of global orbital cutoff., Figure S2: Convergence test image of K-points.
