# Supplementary material for: DFT Analysis of NO Adsorption on the Undoped and Ce-Doped LaCoO3 (011) Surface
Source: Materials (Basel). 2019 Apr 28;12(9):1379. doi: 10.3390/ma12091379 (PMC6540239; doi:10.3390/ma12091379)
Supplement: Supplementary file 1 [file materials-12-01379-s001.pdf]

materials

DFT Analysis of NO Adsorption on the Undoped and Ce-Doped $LaCoO_3$ (011) Surface

Xiaochen Li [1,2] and Hongwei Gao [3,*]

Supplementary materials:

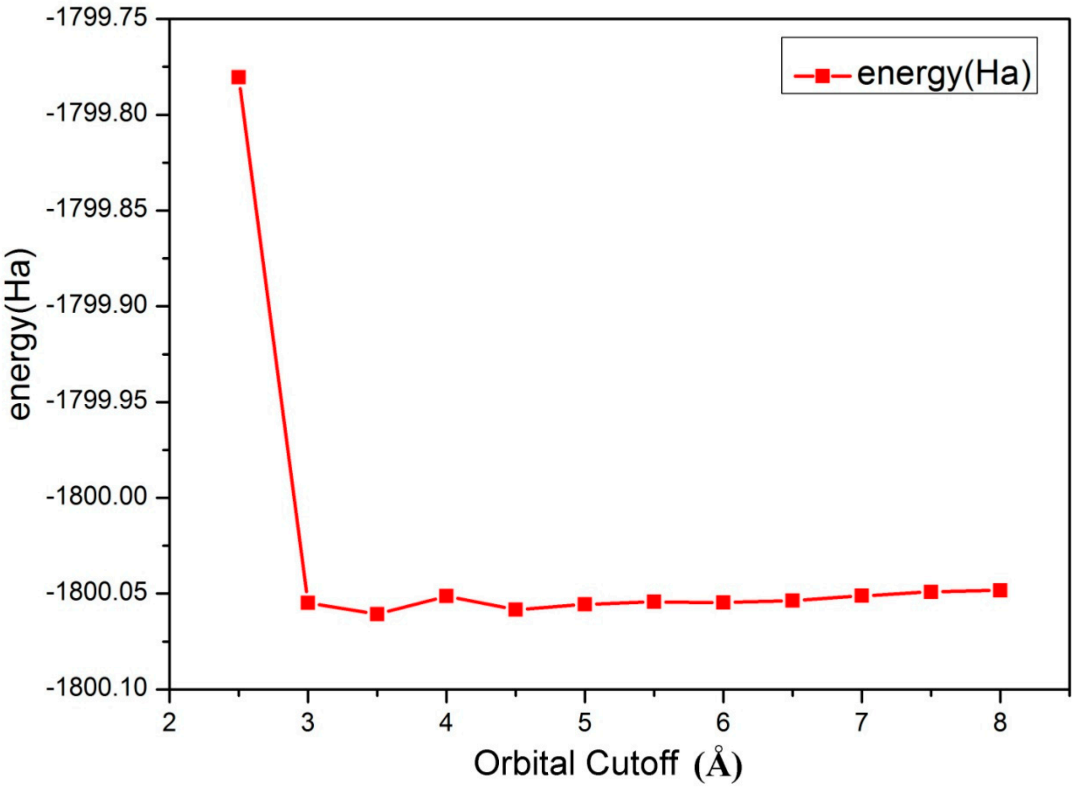

Figure S1. Convergence test image of global orbital cutoff.

materials
MDPI

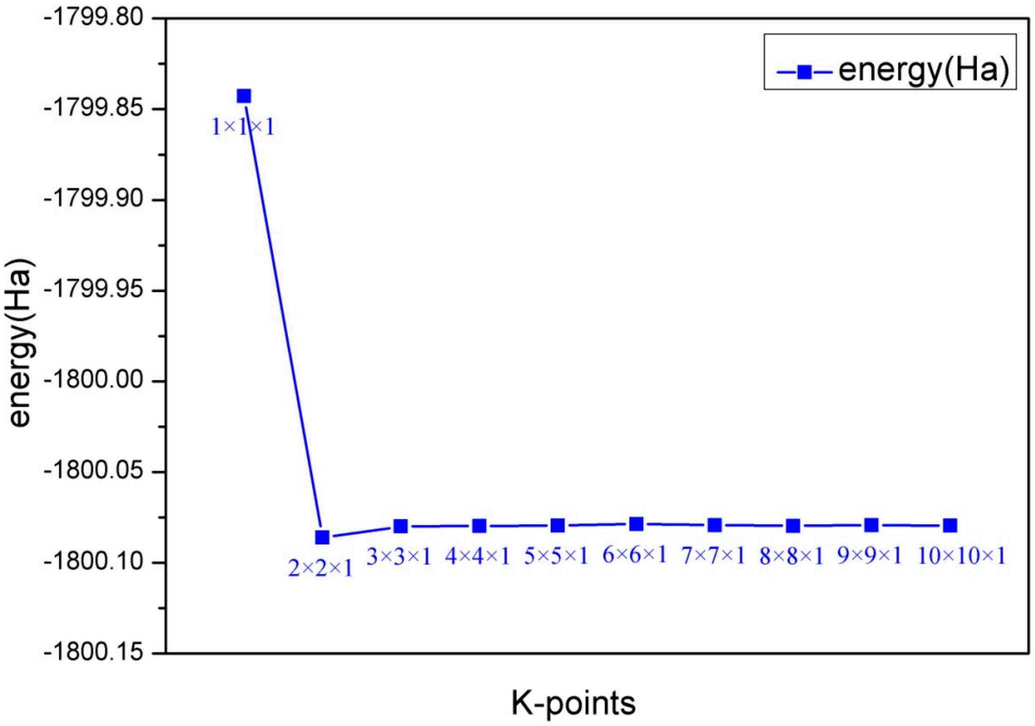

Figure S2. Convergence test image of K-points.
